# Fabrication of Polyurethane–Polyacrylate Hybrid Latexes with High Organosilicon Content via Phase Inversion Emulsion Polymerization

**DOI:** 10.3390/molecules29245870

**Published:** 2024-12-12

**Authors:** Junhao Zhou, Furui Luo, Liming Tang, Zhaoxia Guo

**Affiliations:** Key Laboratory of Advanced Materials of Ministry of Education of China, Department of Chemical Engineering, Tsinghua University, Beijing 100084, China; zjh1717l@163.com (J.Z.); luofurui24@mails.ucas.ac.cn (F.L.); guozx@tsinghua.edu.cn (Z.G.)

**Keywords:** polyurethane–polyacrylate hybrid latex, high organosilicon content, 3-(methacryloyloxy) propyltrimethoxysilane, phase inversion emulsion polymerization

## Abstract

Waterborne polyurethane, with a mechanical strength comparable to solvent-based types, is eco-friendly and safe, using water as a dispersion medium. Polyacrylate excels in film formation and weather resistance but suffers from “hot stickiness and cold brittleness”. Merging polyurethane and polyacrylate creates advanced hybrids, while organosilicon enhances properties but is restricted due to hydrolytic crosslinking. In this paper, a series of polyurethane–polyacrylate hybrid latexes with high organosilicon content were prepared using phase inversion emulsion polymerization technology. Even when the monomer content of 3-(methacryloyloxy)propyltrimethoxysilane (MPS) was increased to 10%, the polymerization process was stable, without the formation of a gel precipitate. The resulting latexes could remain stable for at least 6 months without significant changes in the properties of their films. The effects of MPS content on the mechanical and thermal properties of latex films were systematically researched. The study showed that with an increase in MPS dosage, the hardness and elastic modulus of the latex films increased, while the elongation at break and water absorption decreased, together with the increased glass transition temperature and surface hydrophilicity. This work aims to provide new theoretical guidance for the preparation of silicone-modified hybrid latexes, enabling their safe and stable production and storage.

## 1. Introduction

Due to its customizable and tunable property, polyurethane (PU) represents a kind of materials that hold immense promise across a wide range of applications [1], such as rigid or flexible foams, thermoplastics, coatings, adhesives, etc. Waterborne polyurethane (WPU), primarily using water as the dispersion medium, can significantly reduce the release of organic volatile compounds, thereby meeting environmental protection requirements [2]. WPU, which is garnering growing interest across various industries, not only preserves the essential characteristics of conventional PU but also introduces unique attributes such as low viscosity, non-toxicity, cost-effectiveness, and enhanced safety [3]. Polyacrylate (PA) is also a class of widely used polymers made from acrylate monomers. The uses of PAs are very diverse, and some of their application areas include multifunctional coating [4] and membranes [5], waterproof material [6], leather [7], binders [8], and so on. Compared to individual systems, the mechanical and functional property of hybrid systems comprising PU and PA, namely polyurethane–polyacrylate (PUA), offers the ability to easily adjust the mechanical and functional properties while retaining the benefits of both components [9,10,11,12]. In addition, this approach also achieves cost reduction, as PA is generally less expensive [13]. Based on the above reasons, functional coatings, such as self-matting coatings [14], anti-smudge coatings and anti-corrosive coatings [15,16,17] made up of WPU or waterborne PUA (WPUA) have been extensively reported.

A notable distinction between WPU and conventional PU is that the former employs hydrophilic raw materials as chain extenders (usually containing carboxylic acid, sulfonate, and quaternary ammonium salt) which are essential for the successful preparation and emulsification of WPU [18]. However, the hydrophilicity of WPU can lead to weak water resistance, low hardness and mechanical properties compared to the solvent-based counterparts [19,20,21]. And these shortcomings have markedly limited its practical applications and implementations.

Crosslinkers that are able to react with carboxylic acid groups in the PU/PUA molecular backbones at room temperature, such as aziridine, epoxy-silanes and carbodiimide compounds, can be introduced to significantly enhance the waterproof and mechanical performance of films [22]. For example, the crosslinking density of an inner-crosslinking prepolymer (SiWPU-3) comprising polydimethylsiloxane increased considerably to 6.19 × 10^−3^ mol/cm^3^ from 3.86 × 10^−3^ mol/cm^3^ following the addition of 6 wt% waterborne polycarbodiimide, leading to higher tenacity [23].

Currently, many researchers are dedicated to improving the water resistance and mechanical properties of WPUs. Xiong et al. [16] once introduced dehydrated sorbitan monooleate (SP) into the WPU skeleton to modulate the contents of the traditional trifunctional polyol of trimethylolpropane. The SP-modulated WPU-SP demonstrated exceptional wear resistance and mechanical performance, with the maximum tensile strength reaching an impressive 39.19 MPa. Moreover, the water absorption of WPU-SP decreased by up to 45% versus the traditional WPU with trimethylolpropane. Yu et al. developed a waterproof material by crosslinking waterborne PU, which was dual-modified with silicon and fluorine, using trimethylolpropane-tris-(β-N-aziridinyl) propionate as a simple, fast-curing, and cost-effective crosslinking agent. The crosslinked PU film exhibited notably low water absorption, at only 3.48%, and minimal swelling in n-heptane, with a value of 1.02% within this series of PUs [3]. Our group previously developed a novel polyurethane–urea polyacrylate hybrid emulsion by emulsion copolymerization of polyurethane–urea prepolymer and acrylate monomers, including vinyltriethoxysilane. By incorporating waterborne polycarbodiimide as the room temperature crosslinker, the resultant hybrid coatings were endowed with a triple crosslinking structure. As the amount of polycarbodiimide increased, leading to the consumption of the hydrophilic carboxyl groups within the coating, the water resistance significantly improved, along with enhancements in tensile strength [24]. However, these crosslinking agents must be blended with the emulsion before the coating can be applied, resulting in a two-component system. Moreover, the emulsion with added crosslinking agents cannot be stored and must be used quickly within a short period. Additionally, crosslinking agents such as aziridine are also harmful to the environment and human health.

Organosilicon was applied to improve the water-resistance performance and mechanical properties of PU or PUA [20,24,25]. Unfortunately, organosilicon in an aqueous medium suffers from hydrolysis and accompanying condensation crosslinking owing to its active chemical characteristic. The waterborne silicon-containing PUA is prone to experiencing phenomena such as layering, flocculation, aggregation, demulsification, etc. In the process of synthesizing silicone-modified polymer emulsions (such as silicone-acrylic latexes), therefore, it is often necessary to control the hydrolysis rate and extent of organosilicon to maintain latex stability. Common solutions include employing a sterically hindered silicone monomer [26], pH control [27], or using a large amount of emulsifier (or surfactant) [28,29,30], etc. Even so, the addition of organosilicon is often limited and new problems may arise. For example, the emulsifier that remains in the polymer system can lead to a decrease in the mechanical properties of the film or coating. Motivated by the desire to tailor and enhance the properties of PUA films, this study seeks to vastly increase the content of organosilicon without compromising the storage stability of the resulting latex. To achieve this, a stable soap-free emulsion polymerization method is introduced, which eliminates the need for any external emulsifiers. Instead, the method utilizes polymerizable hydrophilic PU chain segments as the sole emulsifying agent, allowing for the production of high-content organosilicon-modified PUA emulsions.

Herein, a PU prepolymer with polymerizable acrylate groups was synthesized by reacting isophorone diisocyanate (IPDI), dimethylol propionic acid (DMPA), and polypropylene glycol (PPG), followed by being end-capped with 2-hydroxypropyl methacrylate (HPMA). The macromolecular emulsifier with hydrophilic DMPA segments could participate in the polymerization with acrylate monomers including MPS and form a protective layer within the latex particle to prevent the formation of precipitates. Different from traditional emulsion polymerization in which pre-emulsification is generally necessary and the monomers must migrate from the monomer droplets to the latex particles through the aqueous phase before polymerization can occur [26,31,32], oleophilic acrylates and PU emulsifier herein were mixed and dispersed in advance, then water was added. The purpose of this is to reduce the time and probability of MPS coming into contact with water molecules, thereby preventing excessive hydrolysis and the condensation of siloxanes. The hybrid PUA latexes produced in this way can remain stable for up to 6 months at room temperature, even with the MPS content increased to 10 wt% and at a 45 wt% solid content. The effect of MPS content on water absorption and the mechanical and thermal performances of the latex films were investigated systematically. The results demonstrated that the water resistance and the mechanical properties of the resultant PUA films could be noticeably strengthened with the MPS content. This work could provide instructions for producing silicon-containing hybrid emulsions with good storage stability and WPUA films or coatings with satisfactory comprehensive properties.

## 2. Results and Discussion

### 2.1. Preparation of PUA Hybrid Latexes

**Scheme 1:**
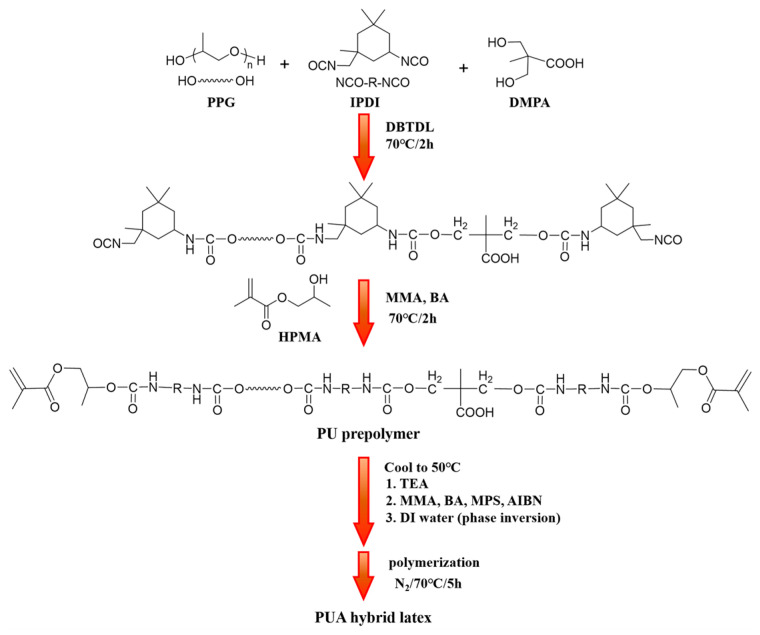
The schematic diagram for synthesizing PUA hybrid latex.

The schematic diagram for synthesizing WPUA latex in this paper is shown in Scheme 1. And the detailed dosage of each raw material is shown in Table 1. In this work, IPDI was reacted with PPG, DMPA, and then the reaction product was terminated with HPMA to form a PU prepolymer with polymerizable acrylate groups. The macromolecular emulsifier polyurethane was thus synthesized via a solvent-free method, only with a small amount of methyl methacrylate (MMA) and n-butyl acrylate (BA) as the diluent. The molar ratio of IPDI, PPG, DMPA and HPMA was settled at 3:1:1:2. Then, the as-obtained prepolymer was neutralized with TEA, and other acrylate monomers and the initiator AIBN were added into the system. Herein, the mass ratio of PU to PA was 2:3. In the PA component, the mass ratio of BA to MMA was fixed at 2:1, and the content of MPS varied from 0 to 10 wt%. Following that, DI water was added to achieve phase inversion under vigorous stirring. Finally, the acrylate-terminated prepolymer copolymerized with MMA, BA and MPS at 70 °C in an inert atmosphere of N_2_, to obtain silicon-containing PUA hybrid latexes. After polymerization, no precipitate was noticed at the bottom of the flask for all the latexes, indicating that the polymerization process was very stable and reliable.

During the phase inversion process, the MPS monomer may contact with water for a short period of time, and a small amount of siloxane groups will inevitably undergo hydrolysis. However, most of the hydrophobic MPS monomers are highly likely to be encapsulated within the interior of the latex particles. After MPS polymerizes to the PA component, its molecular movement is essentially confined within the interior of the latex particles, making it isolated from water and thus hydrolysis occurring at an extremely slow rate. Furthermore, even though MPS may experience hydrolysis and crosslinking, it mostly occurs within individual latex particles. When the latex was coated on a substrate, the latex particles began to coalesce together as the water evaporated. This process would eventually lead to the formation of a continuous polymer film. During this course, the reactive methoxy groups of MPS units in polymer chains could be fully hydrolyzed by water to generate abundant silanol groups, which further underwent self-condensation to form –Si–O–Si– linkages, thus forming a crosslinked network structure for the film [33]. Additionally, even after film formation, the residual siloxanes may undergo further hydrolysis and crosslinking under the action of moisture in the air.

To evaluate the monomer conversion of each group of latex, approximately 2 g of latex was coated on a glass substrate and after it was completely dried, the mass of the resulting film was weighted, which was exactly 45% of the latex mass, corresponding to the theoretical solid content. Therefore, the conversion can be considered nearly 100%, showing that the polymerization process was very efficient.

**Figure 1:**
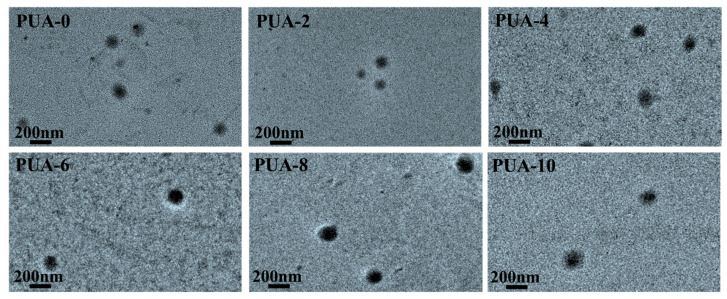
TEM images of different latex particles.

**Figure 2:**
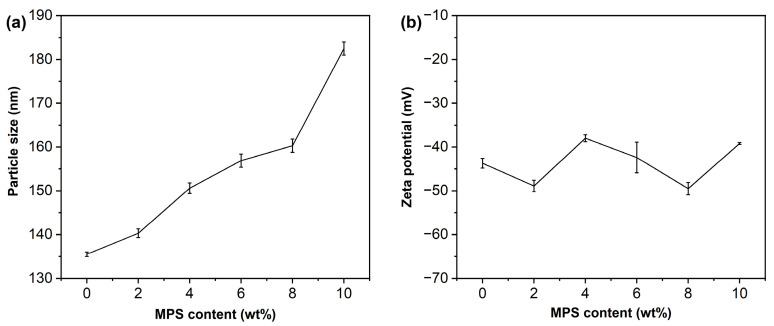
(**a**) Z-average particle size, (**b**) zeta potential of PUA latexes with different MPS content.

TEM images of different latex particles are displayed in Figure 1. The latex particles were generally spherical, and their diameters ranged from about 100 to 200 nm. The particles containing MPS seemed to have a slightly larger size compared to PUA-0, which could be proved by Figure 2a. Based on the FT-IR spectra of the PUA latex films (Figure S1), all films exhibited a strong peak at 1728 cm^−1^ attributed to esteric C=O groups, the wide peak around 3370 cm^−1^ was assigned to -NH of the urethane groups. Unfortunately, for PUA films containing MPS units, the peaks corresponding to Si-O-Si stretching (1000–1100 cm^−1^) or Si-OH stretching (910 cm^−1^) could not be observed [34]. The peaks for those typical groups might overlap with the peaks of other groups, making them indistinguishable.

According to the data by Zetasizer Nano ZS90 equipment (Malvern Instruments, Malvern, UK), as the dosage of MPS increased, the particle size of latexes continuously increased (Figure 2a). PUA-0 had a particle size of 135.5 nm, whereas the particle size was markedly elevated to 182.5 nm for PUA-10. The increasing particle size may result from the small and inevitable hydrolysis condensation of MPS units during the preparation of the latexes. Even if the size of the latex particles increased, however, there would not be an excessive number of collisions or coalescence between them, still resulting in stable latexes. The polydispersity index (PDI) of those latexes from PUA-0 to PUA-10 was 0.113, 0.145, 0.056, 0.050, 0.114, 0.065, respectively, implying that the size distribution of the latex particles was relatively uniform. All latexes possessed a zeta potential ranging from about −40 to −50 mV (Figure 2b), and this rather high negative potential was capable of affording the latexes enough stability.

### 2.2. Latex Stability

The newly prepared latexes had a low viscosity and milky appearance (Figure S2). Even though they were stored for 6 months at room temperature, no phenomena like layering or thickening occurred. Over a period of 6 months, the viscosity of the latexes remained virtually unchanged (Table 2 and Table S1). Additionally, no precipitates were visibly observed at the bottom of the containers. As for the film-forming properties of the latexes, taking PUA-0 and PUA-10 as examples, it can be observed that both newly made and 6-month-stored latexes can form a complete and transparent coating when coated on a glass substrate (Figure S3). These results indicated that the latexes were considerably stable, meanwhile, proving that the scheme of synthesizing WPUA with high silicon content in this work was reasonable and feasible.

**Figure 3:**
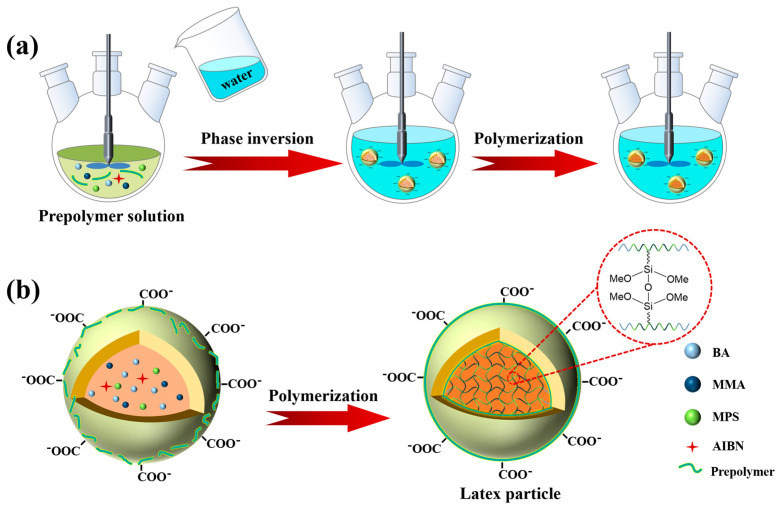
(**a**) Schematic diagram of the phase inversion emulsion polymerization; (**b**) polymerization within a single latex particle.

The novelty of this work lies in the fact that the acrylate monomers and initiator were added to the system in advance and mixed evenly, followed by the addition of water to achieve phase inversion (Figure 3a), during which acrylic monomers were encapsulated in the interior of particles, with a PU emulsifier forming the protective layer. It should be noted that the particles obtained in this work do not possess a traditional core–shell structure, and this point can be proved by Figure 1. Since WPU is amphiphilic, its hydrophilic segments tend to concentrate on the outer surface of the particles, while its hydrophobic segments are more likely to be encapsulated within the particle interior. Therefore, it is likely that the PU component permeates the entire structure of the latex particle. During the phase inversion process, although water may come into contact with the organosilicon monomer, the reaction rate at this temperature (50 °C) was relatively slow. Subsequently, the polymerization was conducted within each particle only, initiated by the generation of free radicals from the thermal decomposition of AIBN at 70 °C (Figure 3b). The advantage of this in situ polymerization approach was that the acrylate monomers could be encapsulated more rapidly within the PU protective layer of the latex particles, reducing the likelihood and duration of contact between MPS and water molecules. As a result, MPS remained stably bound within the hydrophobic interior of the latex particles until the latex film formed. The possibility of Si-OH groups occurring on the surface of the latex particles was thus greatly reduced, preventing collision and coalescence between different particles, as well as demulsification. By contrast, in the traditional emulsion polymerization mechanism, surfactants are generally indispensable to disperse monomers and prevent coalescence between latex particles. That requires the monomer to migrate from monomer droplets to the aqueous phase, and then to the latex particles in which the polymerization occurs. This migration is inefficient and increases the contact time between the monomer and water. Under high-temperature reaction conditions, hydrolysis of organosilicons is inevitable. The resulting hydrophilic silanol structures on the surface of latex particles will further condense with one another, potentially triggering large-sized particles as precipitates. Therefore, the latex obtained in this work could exhibit excellent storage stability, even though the solid content was up to 45%. A reasonable schematic diagram of this protocol is represented in Figure 3.

### 2.3. Effect of MPS

The water contact angle (WCA) images of different coated glasses are displayed in Figure S4, and the corresponding values are listed in Table 2. PUA-0 had a WCA lower than 90° (about 76.4°), indicating that this type of material possessed a certain hydrophilicity that mainly originated from the -COOH groups in the polymer. As the MPS monomers were incorporated into the matrix, the WCAs of the latex films further decreased to about 60°, suggesting an enhancement in the hydrophilicity of the latex films. During the film formation process, the MPS in the latex particles gradually came into contact with water, undergoing hydrolysis to form Si-OH groups, which were then condensed to form Si-O-Si crosslinked structures. However, it was postulated that the retention of Si-OH groups may be likely to contribute to a more hydrophilic film.

**Figure 4:**
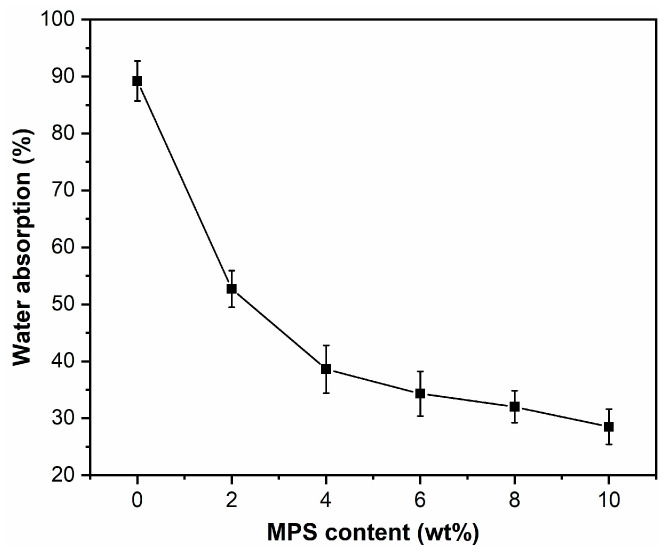
Water absorption of PUA with different MPS contents.

Before incorporating MPS into the PUA matrix, the original PUA film had a water absorption of 89.2 ± 3.5% (Figure 4), and this high value just declared the intrinsic flaw of WPU/WPUA, namely low water resistance. The introduction of MPS could significantly improve the water resistance of those materials. Only 2 wt% MPS could reduce that value to 52.7 ± 3.2%. PUA-10, which had the highest MPS content among the tested samples, demonstrated the greatest waterproofing capabilities, with its water absorption reduced to a mere 28.5 ± 3.1%. The trend in gel content, which rose with increasing MPS content, was in contrast to the pattern observed for water absorption (Table 2). The gel content of PUA-0 was 84.2%, while the value of PUA-10 increased to 88.9%. In the PUA-0 polymer, there was only a copolymerized crosslinked network structure of the acrylate groups from both the PU and acrylate monomers. The results of both decreased water absorption and improved gel content stem from the newly formed Si-O-Si crosslinking structures in the samples.

**Figure 5:**
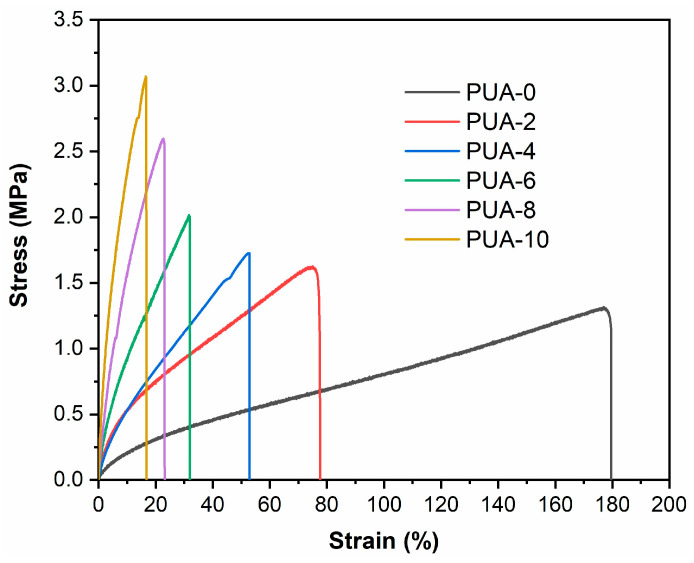
Tensile curves of PUA latex films.

Tensile tests were conducted to evaluate the mechanical performance of PUA latex films and explore the influence of MPS on it. Figure 5 represents typical stress–strain curves of the samples, and their Young’s moduli (E’) are listed in Table 2. Obviously, the introduction of MPS has brought about a significant strengthening effect. E’ of PUA-0 was only 1.6 ± 0.2 MPa, showing it was a relatively soft material (due primarily to the large content of BA monomers in this system, about 40 wt%). PUAs tended to have a higher E’ as the content of MPS rose. From PUA-2 to PUA-10, E’ was enhanced to 7.4 ± 1.4, 7.9 ± 1.3, 13.6 ± 2.9, 21.5 ± 3.8, 45.2 ± 6.6 MPa, respectively, showing the same trend of change as the tensile strength. The tensile strength of PUA-0 was measured at 1.32 ± 0.04 MPa while it was increased to 3.14 ± 0.16 MPa for PUA-10. This continuous mechanical enhancement was mainly attributable to the improved crosslinking density resulting from the hydrolysis and condensation of siloxane groups in polymer chains. More crosslinking structures did indeed restrict the movement of molecular chain segments, which in turn led to a decrease in the elongation at break, and an increased brittleness of this material.

Silane-containing latexes are known for evolving during their storage, even while keeping colloidal stability. Therefore, it is meaningful to investigate if the properties of the PUA films will change over time. The samples, obtained from the latexes after 8 months of storage were subjected to Soxhlet extraction and a tensile test (Figure S5). Their gel contents and E’s were nearly the same as those of the PUA films obtained from fresh latexes (Table S1). In addition, PUA-8 and PUA-10 as representative samples, which were obtained from the fresh latexes and placed under ambient condition for 8 months, were re-tested for tensile curves (Figure S6). There was no significant difference compared with the corresponding PUA films from fresh latexes. These results indicated again that the latexes maintained relatively high stability during storage, and the film properties remained essentially unchanged regardless of when the latex was used to prepare films.

**Figure 6:**
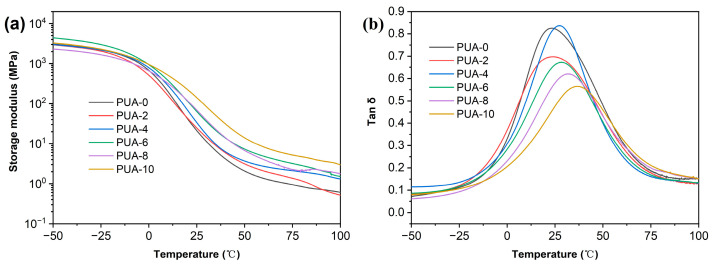
(**a**) Storage moduli and (**b**) loss factors of PUA samples with temperature.

The thermomechanical analysis of the PUA films was investigated by dynamic mechanical analysis (DMA) to acquire their storage moduli (G’) and glass transition temperatures (Tg’s) at which an amorphous polymer transitions from a glassy state to a rubbery state. From Figure 6a, all samples exhibited an initial G’ higher than 2000 MPa at −50 °C, implying a hard, brittle state. G’ would decrease gradually with elevated temperature. That was mainly because the free volume in polymers, namely the empty space between the entangled polymer chains that allows for molecular mobility, began to increase, allowing the polymer chains to start segmental motion. This increased mobility could result in a distinct change in the polymer’s physical properties, such as a decrease in G’ and an increase in thermal expansion and plasticity, exhibiting a softer state. At 25 °C, the G’ exhibited basically a similar trend to tensile strength, indicating that an increase in MPS content correlates with enhanced mechanical performances. Based on the peaks observed in the loss factor (Tan δ) curves, the Tg’s for the samples ranging from PUA-0 to PUA-10, were determined to be 23.2, 23.8, 27.4, 28.3, 31.9, 37.1 °C, respectively (Figure 6b). Free volume would be decreased significantly when more crosslinking points were formed in the polymer three-dimensional network. As for the PUAs, the condensation established by the MPS units in the PA chains caused the polymer chains constrained in their movement, consequently resulting in a higher Tg. Therefore, their Tg’s increased progressively from PUA-0 to PUA-10. In addition, the presence of a single Tg for all samples indicated that PU and PA were bonded together through covalent bonds, which improved the compatibility between the two. However, the Tg’s measured by DSC were not so intuitive as that of DMA, and the corresponding results (Figure S7) are displayed.

All the latexes were coated on a clean tin plate and allowed to dry for a week, thereafter, the properties of the resulting coating were tested. The performances of the latex coatings are listed in Table 3. After the coated tin plates were bent around a 1 mm cylindrical mandrel, all the PUA films did not show any damage or delamination, implying that the films possessed favorable flexibility. The highest adhesion level (0 level) reflected that the coatings formed from the latexes could adhere strongly to some substrates like steel. High impact resistance demonstrated that they possessed some degree of toughness. In terms of pencil hardness, this was elevated as the MPS content increased, which was consistent with the result of the tensile test (Figure 5). In summary, latex coatings could have good performances suitable for practical applications.

**Figure 7:**
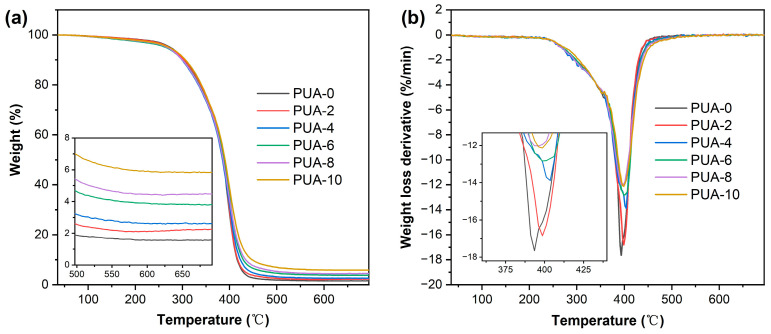
(**a**) TGA curves and (**b**) DTG curves of PUAs.

Coatings or materials are often inevitably applied to settings subjected to intense solar irradiation or other extreme thermal environments, thereby necessitating that they possess enough thermal stability. Thermal stability, which can be assessed by thermogravimetric analysis (TGA), pertains to a material’s capacity to maintain its chemical and physical attributes without significant changes when exposed to elevated temperature conditions. Typical TGA and differential thermogravimetric (DTG) curves of PUA samples are displayed in Figure 7. For PUA-0, the temperature of initial decomposition (T_i_) at which 5% decomposition occurred was 273.4 °C. Other samples exhibited similar T_i_ values. According to DTG curves, only one distinct decomposition peak was revealed. There were no substantial alterations when MPS was introduced to the PUA system. The residual mass of PUA-0 was 1.57%, and the values were 2.24%, 2.62%, 3.80%, 4.47%, and 5.83% when the MPS content was increased to 2, 4, 6, 8, 10 wt%, respectively. This is mainly attributed to the thermotolerant silica formed during the thermal degradation process of –Si–O–Si– linkages. The temperatures at the maximum rate of weight loss (T_max_) had slightly increased. The values of silicon modified PUAs were 398.6 °C, 403.2 °C, 398.7 °C, 393.6 °C, and 399.1 °C, respectively, while the PUA-0 had a corresponding value of 393.5 °C. Overall, the incorporation of MPS did not nearly affect the thermal property of PUAs, and all samples exhibited a degree of thermal stability.

## 3. Materials and Methods

### 3.1. Materials

Dimethylol propionic acid (DMPA, ≥98.0%), triethylamine (TEA, ≥99.0%), isophorone diisocyanate (IPDI, ≥99.0%), azodiisobutyronitrile (AIBN, ≥98.0%) were purchased from Shanghai Merrier Chemical Technology Co., Ltd. (Shanghai, China). Methyl methacrylate (MMA, ≥99.0%), n-butyl acrylate (BA, ≥98.0%) were supplied from Shanghai Macklin Biochemical Technology Co., Ltd. (Shanghai, China). Polypropylene glycol (PPG, Mn = 1000) was procured from Beijing Pusitang Biotechnology Co., Ltd. (Beijing, China), and was vacuum-dried at 100 °C overnight. Di-n-butyltin dilaurate (DBTDL, chemically pure) was obtained from Sinopharm Chemical Reagent Co., Ltd. (Beijing, China). 3-(Methylacryloxy)propyltrimethoxysilane (MPS, ≥99.0%) was supplied by Shanghai Bide Pharmaceutical Technology Co., Ltd. (Shanghai, China). 2-Hydroxypropyl methacrylate (HPMA, 97%) was bought from Tianjin Zancheng Scientific Co., Ltd. (Tianjin, China). Except for PPG, other materials were used without further purification.

### 3.2. Methods

#### 3.2.1. Synthesis of PUA Prepolymer

With mechanical stirring, 6.379 g IPDI, 9.579 g PPG and 1.284 g DMPA, together with 0.1 g DBTDL, were introduced into a three-necked round bottom flask equipped with a condenser tube above which a drying tube was installed. The flask was heated to 70 °C in an oil bath and held for 2 h under the stirring rate of 450 rpm. After that, 2.759 g HPMA along with 5 g MMA and 5 g BA as diluent, was slowly dropped into the flask. The reaction was conducted for another 2 h, giving rise to the corresponding PU prepolymer (20 g). During this period, the setup was open to the outside air to avoid polymerization of the acrylate monomers.

#### 3.2.2. Synthesis of PUA Latex

After the mixture was cooled to about 50 °C, 1.161 g TEA was added to neutralize the carboxylic acid groups in PU prepolymer. According to Table 1, the stoichiometric MPS and the remaining required amount of MMA, BA (totaling 30 g of acrylate monomers) were added dropwise and stirred for 5 min, following which AIBN (1 mol% of acrylates) was added to the flask and stirred to ensure it dissolves completely and evenly within 5 min. Subsequently, after the stirring rate was further increased to 600 rpm, 61.111 g deionized water was added to the flask and vigorously stirred, giving rise to a milky aqueous dispersion. The reactor was then purged with nitrogen for at least 10 min and kept isolated from external atmosphere. Finally, the emulsion polymerization of the macromolecular emulsifier and acrylate monomers was carried out for 5 h at 70 °C, to obtain the PUA hybrid latex with 45% solid content.

#### 3.2.3. Synthesis of PUA Hybrid Film

Drop suitable amount of PUA latex into a homemade tetrafluoroethylene mold (7 × 7 × 0.3 cm^3^) and dry for a week at room temperature. The as-obtained film was peeled off for different characterization. In addition, the latex was applied to clean tinplate or glass sheets and allowed to dry for a week, after which the properties of the resulting coating were tested.

#### 3.2.4. Characterization

The emulsion was firstly diluted to 1/2500 of original concentration, then a Zetasizer Nano ZS90 equipment (Malvern Instruments, Malvern, UK) was used to obtain the particle size and zeta potential. After the emulsion was diluted to an appropriate level, the morphology of emulsion particles was observed by transmission electron microscopy (TEM, JEM-2010, JEOL, Tokyo, Japan).

Fourier transform infrared (FTIR, Shimadzu IR tracer-100, Kyoto, Japan) spectrometer was utilized to record the FTIR spectra of those PUA films. The thermal stability of those films was characterized by a thermogravimetric analyzer (TG 209, NETZSCH Corporation, Selb, Germany) at a heating rate of 10 °C/min with N_2_ flow of 20 mL/min. The heat behavior of PUA films was performed via a differential scanning calorimeter (DSC, DSC 214 Polyma, NETZSCH, Selb, Germany) at a heating rate of 10 K/min. Dynamic mechanical analysis (DMA) was conducted by a TA Instrument Q800 DMA (Waters, New Castle, DE, USA) at a heating rate of 3.0 K/min and frequency of 1 Hz. The coated glasses were used to perform water contact angle (WCA) test, and the data were recorded using a contact angle goniometer (Dataphysics OCA 20, Filderstadt, Germany). A drop of water (5 μL) was dropped onto the hybrid coating at room temperature and every test was repeated three times. The mechanical property of PUA samples with the size of 30 × 7 × 1.5 mm was evaluated by an Instron 5967 testing machine (Instron Co., Ltd., Norwood, MA, USA) at an extension rate of 10 mm/min. Repeat the experiment three times and take the average value.

About 1 g PUA sample was wrapped completely in filter paper and placed into a Soxhlet extractor. The package was Soxhlet extracted with tetrahydrofuran at 90 °C for 12 h. After that, the package was dried in an oven at 70 °C for 24 h, then the sample was taken out and weighted. The gel content was calculated using the following equation:

(1) $$\mathrm{Gel}\;\mathrm{content}=\frac{m_{2}}{m_{1}}\times 100\%$$

where *m*_1_ was the original weight of PUA sample and *m*_2_ was the weight of the residue after Soxhlet extraction.

About 1 g PUA film was soaked in deionized water. After immersion for 24 h, took the sample out, and gently wiped the surface dry with filter paper. The swollen sample was weighted again. Swelling degree was obtained based on the following equation:

(2) $$Swelling\;degree=\frac{m_{4}-m_{3}}{m_{3}}\times 100\%$$

where *m*_4_ and *m*_3_ were the weight of PUA sample after and before soaking, respectively.

The impact resistance of PUA coatings formed on tinplate sheets were assessed by the QCJ-50 film impactor. In the test, a 1.0 kg hammer fell freely on the coated tin plate from different heights (the maximum height is 50.0 cm). The impact resistance referred to the maximum height (h_max_ cm) at which the ball could fall without damaging the coating, expressed as h_max_ kg·cm [35]. Multiple measurements were repeated and the consistent result was taken as the evaluation value. The adhesion grade, pencil hardness, flexibility of the PUA coatings formed on tinplate sheets, obtained from tensile test were conducted according to our previous work [36].

Unless otherwise specified, the characterization described in the text is for films or coatings prepared from fresh latexes.

## 4. Conclusions

This paper presents a new method for preparing stable, high-content organosilicon-modified PUA emulsions and provides a detailed characterization of the emulsions and the corresponding latex films. TEM images showed that the latex particles were nearly spherical, and no obvious coalescence was observed. Particle size analysis indicated that as the amount of MPS increased, the size of the latex particles gradually increased, but all were less than 200 nm. Stability tests showed that even with a high solid content of 45%, the emulsion could be stored stably for 6 months under laboratory conditions and maintained a low viscosity state. The water resistance and mechanical properties of MPS-modified PUA films were significantly improved; compared to PUA-0, the water absorption of PUA-10 was reduced from 89.2 ± 3.5% to 28.5 ± 3.1%, and the Young’s modulus was increased from 1.6 ± 0.2 MPa to 45.2 ± 6.6 MPa, with increasing MPS content to 10%. These results were attributed to the newly formed crosslinking effects of siloxane groups in the PA chain segments. Additionally, the coatings derived from this latex also exhibited good adhesion, toughness, and impact resistance, demonstrating potential for use as coatings with excellent comprehensive performance. Another important point is that the in situ polymerization technique herein can reduce the time and probability of contact between the easy-to-hydrolyze organosilicon and water, thereby avoiding precipitates caused by the hydrolysis and condensation. This technology holds significance for addressing practical needs and theoretical research, and it offers a new approach to designing stable silicone-modified PUA or silicone–acrylic emulsions. It is foreseeable that this polymerization method can also be extended to other waterborne polymer systems formed via phase inversion emulsion polymerization, such as polyacrylates, epoxy–acrylate hybrid resins, and so on, providing a new route for the preparation of waterborne polymers with high silicone content.

## Figures and Tables

**Table 1:** Feeding formula for preparing PUA hybrid latexes.

Sample ^1^	PU (g)	MMA (g)	BA (g)	MPS (g)	AIBN (g)
PUA-0	20	10.00	20.00	0	0.452
PUA-2	20	9.67	19.33	1	0.444
PUA-4	20	9.33	18.67	2	0.437
PUA-6	20	9.00	18.00	3	0.429
PUA-8	20	8.67	17.33	4	0.422
PUA-10	20	8.33	16.67	5	0.415

^1^ For PUA-n, the number n represents the mass percentage of MPS in PUA is n%.

**Table 2:** The viscosity of the latexes and the performance parameters of the latex films.

Sample	Viscosity (mPa·s)	WCA (°)	Gel Content (%)	Young’s Modulus (MPa)	Tg (°C)
PUA-0	47	76.4 ± 6.2	84.2	1.6 ± 0.2	23.2
PUA-2	52	59.0 ± 5.7	85.1	7.4 ± 1.4	23.8
PUA-4	40	63.6 ± 7.8	85.3	7.9 ± 1.3	27.4
PUA-6	46	60.6 ± 5.8	86	13.6 ± 2.9	28.3
PUA-8	39	60.4 ± 3.6	88.6	21.5 ± 3.8	31.9
PUA-10	68	63.8 ± 4.1	88.9	45.2 ± 6.6	37.1

**Table 3:** The performances of the latex coatings.

Sample	Flexibility (mm)	Adhesion Level	Impact Resistance (Kg·cm)	Pencil Hardness
PUA-0	1	0	50	B
PUA-2	1	0	50	HB
PUA-4	1	0	50	H
PUA-6	1	0	50	2H
PUA-8	1	0	50	2H
PUA-10	1	0	40	3H

## Data Availability

Data in this paper can be obtained from the corresponding author.

## Supplementary Materials

The following supporting information can be downloaded at: https://www.mdpi.com/article/10.3390/molecules29245870/s1, Figure S1: FT-IR spectra of PUA films; Figure S2: Appearance of (a) newly prepared and (b) 6-month stored latexes; Table S1: The viscosity of the latexes, the gel content and Young’s modulus of the latex films. Figure S3: Films coated on glass from (a) newly prepared and (b) 6-month stored emulsions. Figure S4: Water contact angle images on different PUA films; Figure S5: Tensile curves of PUA films from the latexes after being stored for 8 months. Figure S6: Tensile curves of PUA-8 and PUA-10 films after being placed under ambient conditions for 8 months. Figure S7: DSC curves of PUA samples.
