# Supplementary material for: Fabrication of Polyurethane–Polyacrylate Hybrid Latexes with High Organosilicon Content via Phase Inversion Emulsion Polymerization
Source: Molecules. 2024 Dec 12;29(24):5870. doi: 10.3390/molecules29245870 (PMC11678795; doi:10.3390/molecules29245870)
Supplement: Supplementary file 1 [file molecules-29-05870-s001.zip › molecules-3317206-supplementary.pdf]

Supporting Information

Fabrication of polyurethane-polyacrylate hybrid latexes with high organosilicon content via phase inversion emulsion polymerization

Junhao Zhou, Furui Luo, Liming Tang *, and Zhaoxia Guo

Key Laboratory of Advanced Materials of Ministry of Education of China, Department of Chemical Engineering, Tsinghua University, Beijing 100084, China;

* Correspondence: tanglm@tsinghua.edu.cn (L.T.)

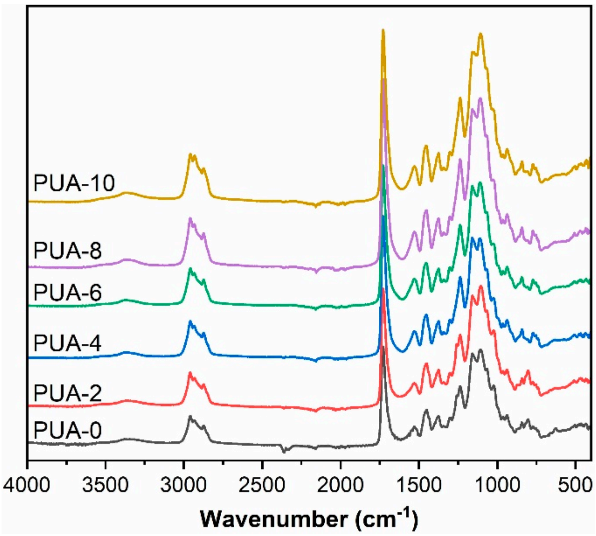

Figure S1. FT-IR spectra of PUA films.

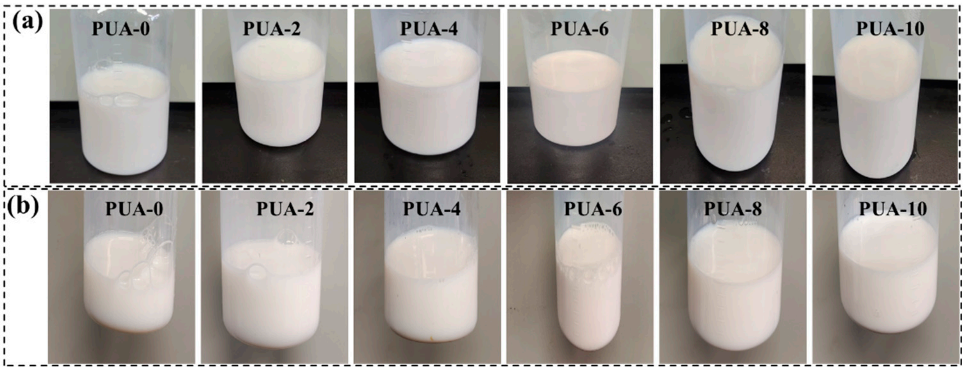

Figure S2. Appearance of (a) newly prepared and (b) 6-month stored latexes.

Table S1. The viscosity of the latexes, the gel content and Young's modulus of the latex films.

Sample	Viscosity [1] (mPa·s)	Gel content [2] (%)	Young's modulus [3] (MPa)
PUA-0	49	84.7	1.8±0.4
PUA-2	50	85.4	6.5±0.9
PUA-4	44	85.9	10.8±1.0
PUA-6	42	86.4	17.3±3.8
PUA-8	47	88.2	24.3±3.1
PUA-10	61	88.6	48.7±7.2

[1] The viscosity of latexes after storing for 6 months. [2] Gel content and [3] Young's modulus of the films from latexes after being stored for 8 months.

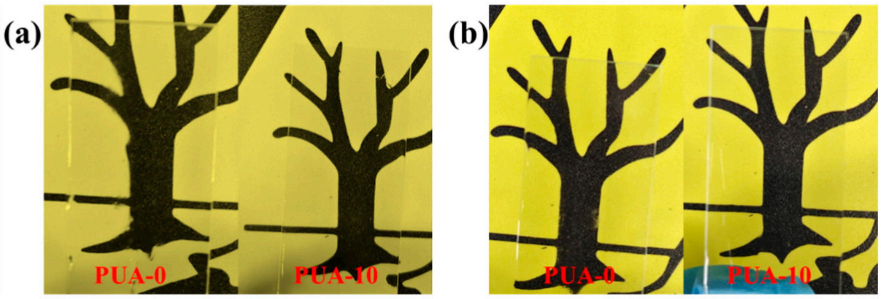

Figure S3. Films coated on glass from (a) newly prepared and (b) 6-month stored latexes.

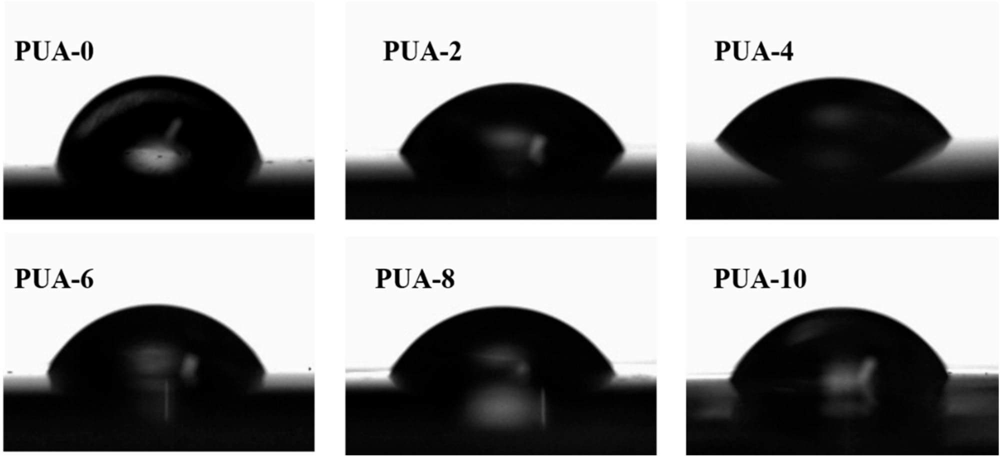

Figure S4. Water contact angle images on different PUA films.

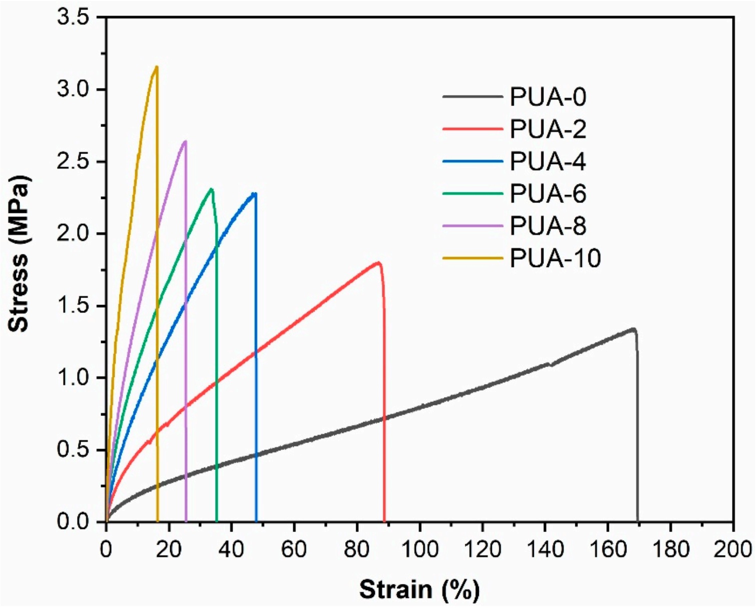

Figure S5. Tensile testing curves of PUA films from the latexes after being stored for 8 months.

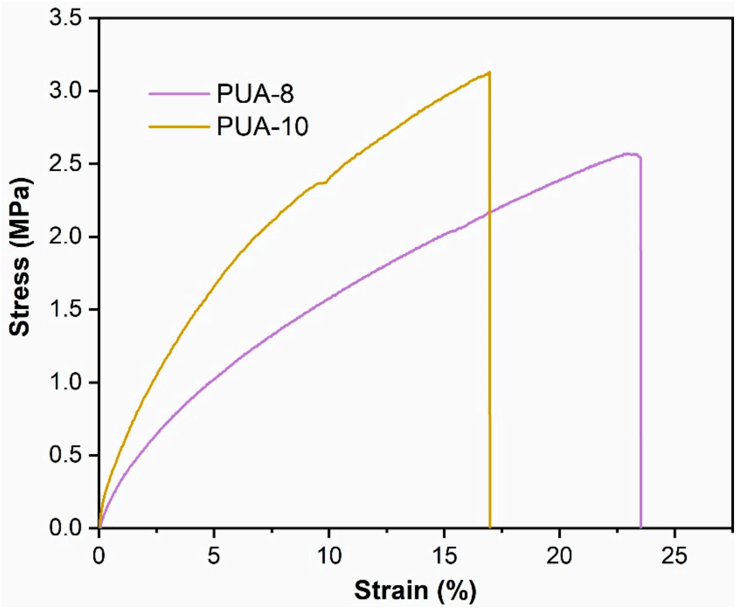

Figure S6. Tensile testing curves of PUA-8 and PUA-10 films after being placed under ambient condition for 8 months.

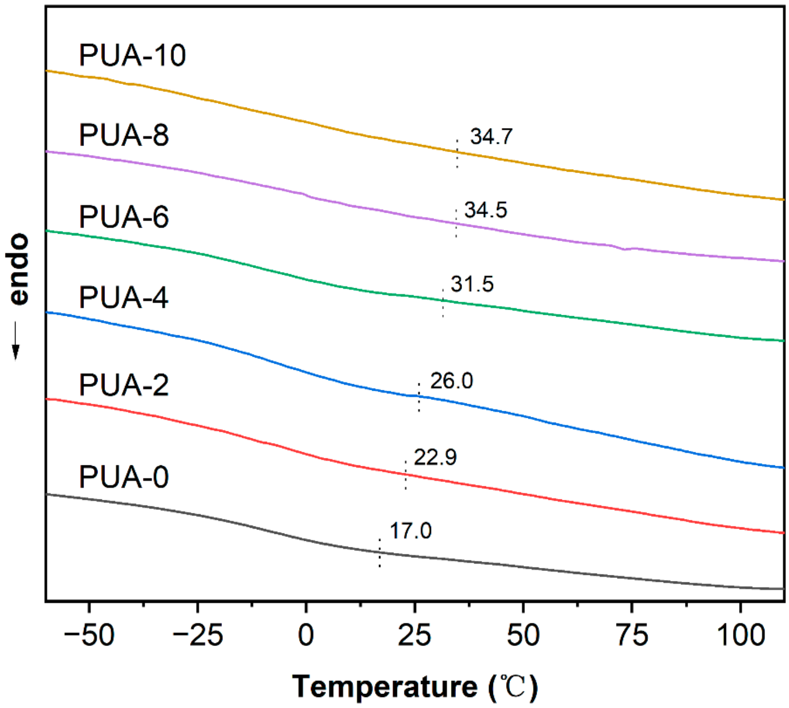

Figure S7. DSC curves of PUA samples.
